# SDHB-Associated Paraganglioma in a Pediatric Patient and Literature Review on Hereditary Pheochromocytoma-Paraganglioma Syndromes

**DOI:** 10.1155/2014/502734

**Published:** 2014-09-15

**Authors:** Heather Choat, Kerri Derrevere, Lisa Knight, Whitney Brown, Elizabeth H. Mack

**Affiliations:** ^1^Department of Pediatrics, University of South Carolina School of Medicine, Columbia, SC 29203, USA; ^2^Division of Pediatric Endocrinology, Department of Pediatrics, University of South Carolina School of Medicine, 9 Medical Park Drive, Suite 230A, Columbia, SC 29203, USA; ^3^Division of Pediatric Critical Care, Palmetto Health Children's Hospital, Columbia, SC 29203, USA

## Abstract

Pheochromocytoma and paraganglioma are rare in the pediatric population occurring in approximately 1 in 50,000 children. While some cases are sporadic, they have commonly been associated with syndromes such as von Hippel-Lindau, multiple endocrine neoplasia types IIa and IIb, neurofibromatosis type 1, and hereditary pheochromocytoma-paraganglioma syndromes. In children less than 18 years of age approximately 60% of pheochromocytomas and paragangliomas are associated with a germline mutation. We present an 11-year-old child with an abdominal paraganglioma related to a succinate dehydrogenase subunit B gene mutation whose father had a previously resected abdominal paraganglioma and was found to carry the same mutation. In addition, we review the etiology, genetics, diagnostic approach, and challenges of preoperative management of secretory pheochromocytomas and paragangliomas in children.

## 1. Introduction

Pheochromocytomas (PCC) and paragangliomas (PGL) are rare tumors in children. PCC are neuroendocrine tumors located within the adrenal gland that arise from chromaffin cells of neural crest origin and commonly produce one or more catecholamines. PGL comprise an extra-adrenal subset of PCC arising from either sympathetic or parasympathetic paraganglia [1–3]. Approximately 10–20% of all PCC/PGL are diagnosed in children with an average age at diagnosis of 11 years [4, 5]. It has been reported that over half of all PCC and PGL in patients <18 years old are associated with a germline mutation and that this percentage seems to increase with decreasing age at diagnosis [6]. They have been commonly associated with heritable conditions such as multiple endocrine neoplasia (MEN) types IIa and IIb, von Hippel-Lindau, and hereditary PCC/PGL syndromes [1–6]. In general, PGL located in the head and neck are more often parasympathetic and nonsecretory whereas PGL within the abdomen are more likely to be sympathetic and are associated with catecholamine secretion [1, 2].

## 2. Case Report

**Figure 1:**
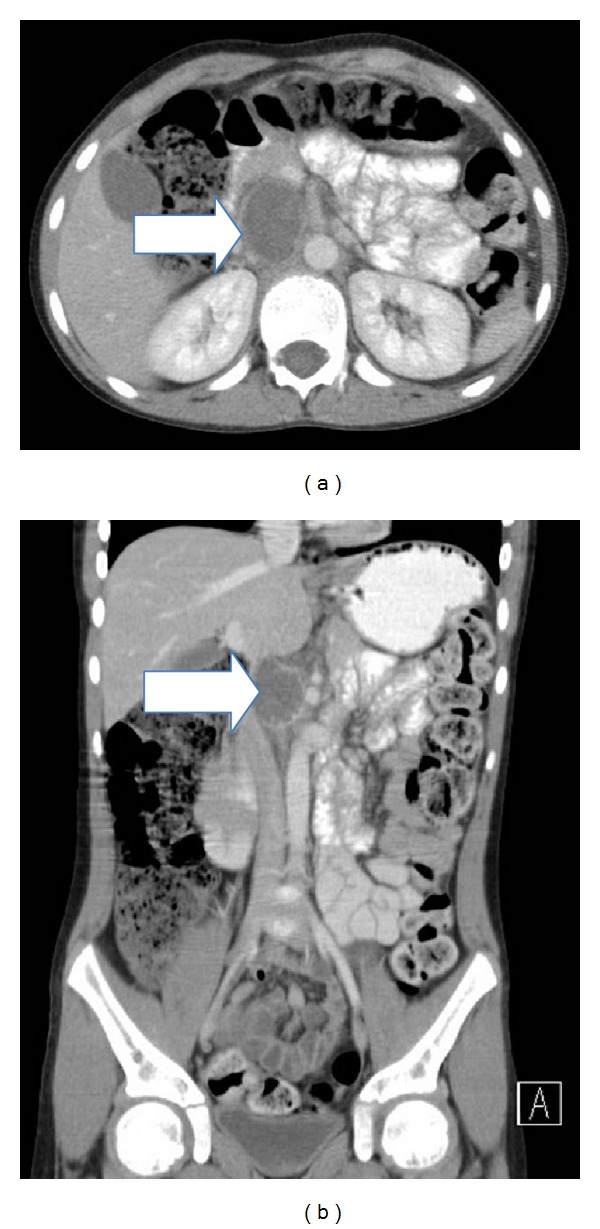
Axial (a) and coronal (b) images of the 3 × 2.3 cm retroperitoneal mass between aorta and inferior vena cava.

A previously healthy 11-year-old Caucasian male presented with a chief complaint of periumbilical abdominal pain for one day. He had multiple episodes of nonbloody, nonbilious emesis and decreased oral intake for one day prior to presentation, but no associated fever or diarrhea. Notably he had a two-month history of headaches upon awakening occurring two to three times per week. On presentation, his heart rate was 136 beats per minute and his blood pressure was 160/120 mmHg. An abdominal computed tomography (CT) scan revealed a 3 cm by 2.3 cm retroperitoneal mass between the inferior vena cava and aorta (Figure 1). He was transferred to the pediatric intensive care unit (PICU) for further management of the retroperitoneal mass and hypertensive crisis.

On exam he was nontoxic and no flushing of the skin was present. His abdomen was soft with normal bowel sounds and without palpable masses, guarding, or rebound tenderness. Further questioning revealed that the patient's father underwent resection of abdominal PGL requiring preoperative antihypertensive therapy and postoperative radiation at the age of 24 years. The PGL was found incidentally on imaging performed after a motor vehicle collision. After the patient's diagnosis, further history revealed that the paternal grandfather and a maternal cousin were also diagnosed with PCC. Diagnostic evaluation showed polycythemia with hemoglobin of 17 g/dL, normal echocardiogram, normal electrolytes, and normal renal function.

In the PICU, the patient's hypertension was initially treated with isradipine but was still associated with intermittent tachycardia. Vanillylmandelic acid (VMA) and plasma normetanephrine levels returned elevated, indicating a neuroendocrine secretory tumor. On the third day *α*-adrenergic blockade was initiated with phenoxybenzamine 0.2 mg/kg/dose orally twice daily. Forty-eight hours later he was started on *β*-blockade with propranolol 0.15 mg/kg/dose orally twice daily.

Despite gradual increases in *α*- and *β*-blockade with phenoxybenzamine, atenolol, and propranolol, he ultimately required an esmolol infusion to maintain the heart rate less than 100 beats per minute. In order to reduce the likelihood of intraoperative and postoperative morbidity, phenoxybenzamine was discontinued 48 hours prior to surgery, and *β*-blockade was stopped 8 hours prior to surgery. He received *α*- and *β*-blockade for 23 days prior to resection.

**Figure 2:**
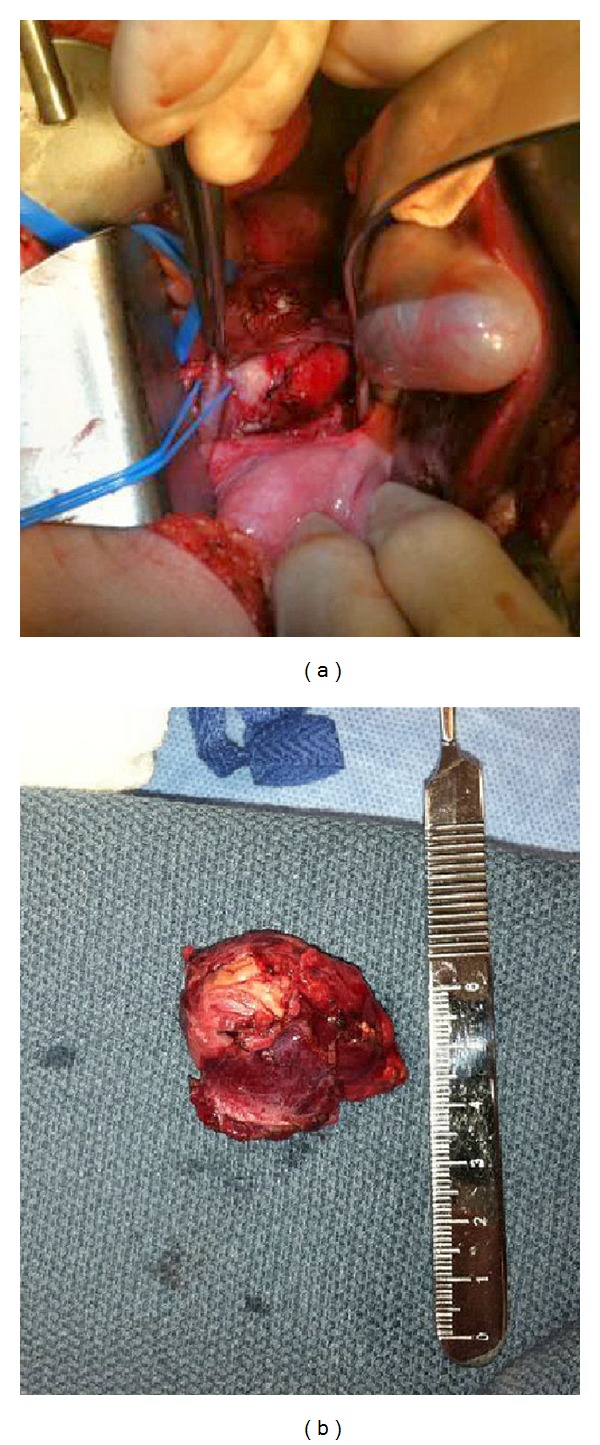
Intraoperative (a) and postoperative (b) views of the retroperitoneal mass.

**Figure 3:**
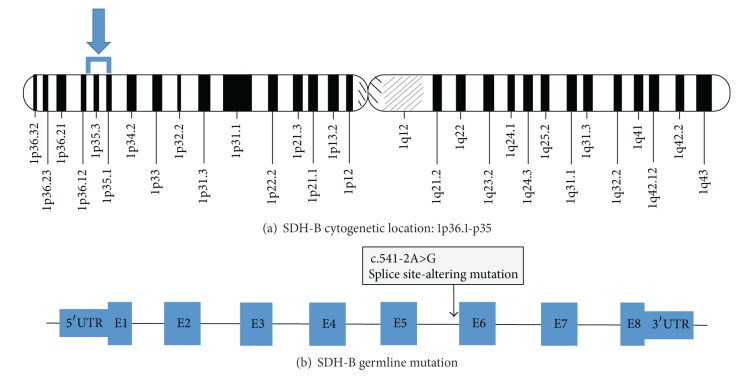
Chromosomal location of the SDHB gene (a) and site of the patient's specific mutation (b).

Laboratory evaluation revealed elevations in the following: plasma normetanephrine 1.72 nmol/L (normal 0–0.89), urine normetanephrine 799 *μ*mol/mol creatinine (normal 0–278), and VMA 15.1 mg/g creatinine (normal <8.6). Due to the location of the retroperitoneal mass, the patient was not a candidate for laparoscopic approach for its removal. The mass was resected on hospital day 23, and the procedure was uncomplicated (Figure 2). Pathology confirmed the diagnosis of paraganglioma. Following resection, the patient remained normotensive and was discharged home four days postoperatively off all antihypertensive medications. After discharge, a serum genetic panel returned positive for a heterozygous deleterious mutation in the succinate dehydrogenase complex subunit B (SDHB) gene. This gene, located at 1p.36.1-1p.35, was PCR amplified and then sequenced in the forward and reverse directions using automated fluorescent dideoxy sequencing methods. The patient's specific mutation was the splice site-altering change c.541-2A>G located in intron 5 of the gene (Figure 3).

**Figure 4:**
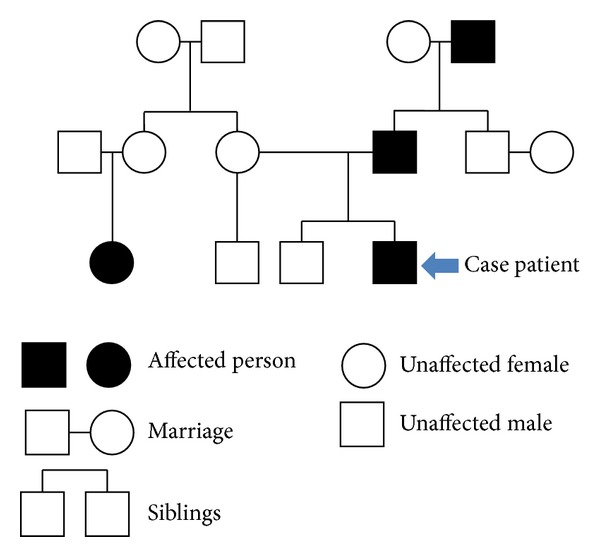
Family pedigree of individuals affected with PCC/PGL.

In the pediatric endocrinology clinic two weeks after discharge, the patient was asymptomatic and normotensive. Repeat plasma normetanephrine at that time was decreased but still mildly elevated at 1.15 nmol/L (normal 0–0.89). His family was referred for genetic counseling at this time. The patient's father was found to carry the same genetic mutation, and his younger brother's genetic panel returned normal. Incidentally, a female maternal cousin had previously been found to have a PCC, but she neither did undergo genetic testing nor did the paternal grandfather (Figure 4). One month postoperatively, the patient was seen for follow-up at the National Institutes of Health (NIH) where he had a CT of the neck, chest, abdomen, and pelvis which showed no evidence of residual or metastatic disease. Also, at that time, laboratory assessment showed normal catecholamine levels including urine epinephrine, urine norepinephrine, urine dopamine, urine metanephrines, urine normetanephrines, and total urine metanephrines.

Five months postoperatively, the patient was seen again in the pediatric endocrinology clinic and continued to be asymptomatic and normotensive. Eight months postoperatively at the NIH, magnetic resonance imaging (MRI) of the neck, chest, abdomen, and pelvis showed no evidence of PGL. Laboratory assessment including 24-hour urine epinephrine, norepinephrine, dopamine, metanephrine, normetanephrine, total metanephrines, plasma epinephrine, norepinephrine, and dopamine levels was all within normal range. Thyroid studies at that time were also normal.

Twenty months postoperatively, he was again seen at the NIH and had an unremarkable physical exam and was asymptomatic and normotensive. Repeat MRI of the neck, chest, abdomen, and pelvis showed no evidence of local or metastatic tumor recurrence. Plasma and urine catecholamines and chromogranin A were again found to be in normal ranges. He is now being followed on an annual basis.

## 3. Discussion

Our patient presented with many of the typical signs and symptoms of PCC/PGL, most notably with new onset secondary hypertension. With the high incidence of childhood overweight and obesity in the United States (occurring in about 30% of children) the leading cause of pediatric hypertension continues to be essential hypertension, whereas PCC/PGL account for only about 1% of cases [7]. Causes of secondary hypertension in the pediatric population are more commonly associated with certain medications, renal disease, renovascular disease, Cushing syndrome, or hyperthyroidism. However, as our case clearly demonstrates, rare causes including PCC or PGL should always be considered in the setting of secondary hypertension [2, 4, 6].

Diagnostic evaluation for PCC/PGL begins with biochemical evaluation followed by anatomic imaging, with functional imaging if metastatic disease is present, and surgical pathology (Table 1). The tests of choice for biochemical evaluation are plasma free metanephrines and/or fractionated urine metanephrines and normetanephrines, which have a sensitivity approaching 100% for diagnosis of a catecholamine-secreting tumor [1, 8–11]. Chromogranin A is another biochemical marker that correlates with tumor size, malignant potential, and is a marker for the rare SDHB-related PGL [1, 12]. In addition, patients may have hyperglycemia, increased erythrocyte sedimentation rate, polycythemia, and leukocytosis on preliminary screening. Initial anatomic imaging should include CT of the abdomen and pelvis and then CT of the neck and chest if initial imaging is negative [1, 8]. CT is recommended as the first-choice imaging modality rather than MRI except in cases of known metastatic disease, for detection of skull base and neck PGL, in patients with surgical clips causing image artifact when using CT, in CT contrast-allergic patients, and in patients in whom radiation exposure should be limited such as children, pregnant women, patients with known germline mutations, and those with excessive radiation exposure [13]. For metastatic disease or patients in whom catecholamines are borderline elevated, functional imaging can provide additional diagnostic clarification. Previously, nuclear scintigraphy imaging with ^123^I-labeled metaiodobenzylguanidine (MIBG) was common but has been proven to be less sensitive than newer modalities such as ^18^F-fluorodihydroxyphenylalanine or ^18^F-fluorodopamine positron emission tomography (PET) particularly in PGL and metastatic disease [14]. In cases of PCC and PGL, malignancy refers to metastases in locations where paraganglia are not usually found such as bone, lung, and liver.

Definitive treatment of secretory PCC/PGL is surgical resection; however, preoperative medical management of hypertension is equally important to ensure good clinical outcomes. The mainstay of therapy is to first initiate nonselective α-adrenergic blockade with phenoxybenzamine. Once α-blockade has been accomplished, *β*-blockade is initiated with either a nonselective or a *β*1-selective blocker such as propranolol or metoprolol, respectively. *β*-Blockade should not be used initially or as a single agent due to resultant reflex tachycardia and worsening hypertension from unopposed *α*-adrenergic activation. Treatment should be initiated 1-2 weeks prior to resection to avoid complications from intraoperative catecholamine surges [5, 15, 16]. Also, 1-2 days preoperatively, *α*-blockade should be stopped and patients may be salt-loaded to avoid postoperative hypotension. Surgical resection is most commonly achieved laparoscopically, but with large tumors or concern for malignancy a laparotomy may be performed [1, 17, 18].

Although some cases of pediatric PCC/PGL are sporadic, it has been reported that 59% of those occurring in children <18 years old and up to 70% of those occurring in children <10 years old are associated with a germline mutation [6]. Syndromes commonly linked to PCC/PGL are von Hippel-Lindau, neurofibromatosis type I, MEN IIa and IIb, and hereditary PCC/PGL syndromes. In the case of our patient and his father, genetic testing revealed the same deleterious mutation in SDHB, reinforcing the diagnosis of a familial paraganglioma syndrome. This particular mutation is not novel, and it is one of over 100 SDH gene mutations which have been reported [19]. The SDH enzyme complex is made up of several subunits (SDHA, SDHB, SDHC, SDHD, and SDH assembly factor 2) and is integral in linking the tricarboxylic acid (TCA) cycle to the process of oxidative phosphorylation within mitochondria. Specifically, SDHB is a known tumor suppressor gene which functions to prevent the accumulation of toxic metabolites during the TCA cycle. While SDH subunit gene mutations have been shown to be associated with neoplasia and cause susceptibility to familial PCC/PGL syndromes, an exact causal mechanism is still unclear [20]. It is widely proposed, however, that the affected subunit determines the phenotype [3].

Hereditary PCC/PGL syndromes have an autosomal dominant mode of inheritance, and approximately 40% of patients with SDHB mutations may go on to develop malignant disease [13, 15, 20]. Overall SDHB mutations are associated with a higher malignancy rate than mutations in the SDHD or SDHC genes and in fact account for 50% or more of malignant PCC and PGL [1]. SDHB mutations usually correlate with disease in the thorax, abdomen, or pelvis while SDHD mutations often manifest as head or neck tumors that are typically benign [6, 12, 17]. A task force selected by the Endocrine Society recently released new practice guidelines recommending that all patients with PCC or PGL be engaged in a shared decision-making process regarding genetic testing [13]. Routine genetic counseling and testing are recommended, however, after the diagnosis of PGL in a child regardless of family history particularly since certain mutations can indicate multisite disease or higher probability of malignancy [17, 21]. Metastatic disease carries a poorer prognosis; 5-year survival probability decreases from 89% in nonmalignant cases to 20–70% in malignant cases depending on location [3]. Therefore, it is important to accurately distinguish between benign and metastatic diseases at the time of diagnosis to ensure sufficient follow-up and treatment. It is currently recommended that mutation-carrying patients who are tumor-free be followed on an annual basis [13]. While a TCA cycle gene mutation database has been created, data are lacking to determine accurate recurrence risk for hereditary PCC and PGL, particularly those diagnosed in children. Research focused in this area is needed to further guide follow-up recommendations and optimize long-term outcomes in the future.

## Figures and Tables

**Table 1:** Work-up of suspected PCC/PGL.

Biochemical testing	
Fractionated urine metanephrines	
Fractionated plasma free metanephrines	
Chromogranin A	

Anatomic imaging	
Ultrasound (children)	
CT	
MRI	
Functional imaging	
^18^F-Fluorodihydroxyphenylalanine PET	
^18^F-Fluorodopamine PET	
^123^I-MIBG single-positron emission CT	
